# Circular RNA ITCH Is a Tumor Suppressor in Clear Cell Renal Cell Carcinoma Metastasis through miR-106b-5p/PDCD4 Axis

**DOI:** 10.1155/2021/5524344

**Published:** 2021-04-20

**Authors:** Ping Gao, Yong Huang, Yanmei Hou, Qian Li, Haimei Wang

**Affiliations:** ^1^Department of Urology Surgery, Hospital of Chengdu University of Traditional Chinese Medicine, No. 39 Shi-er-qiao Road, Chengdu, 610072 Sichuan Province, China; ^2^Department of Pharmacy, Hospital of Chengdu University of Traditional Chinese Medicine, No. 39 Shi-er-qiao Road, Chengdu, 610072 Sichuan Province, China; ^3^Department of Proctology, Hospital of Chengdu University of Traditional Chinese Medicine, No. 39 Shi-er-qiao Road, Chengdu, 610072 Sichuan Province, China; ^4^Department of Urology, Huai'an Second People's Hospital and The Affiliated Huai'an Hospital of Xuzhou Medical University, Huai'an, China

## Abstract

High metastasis of clear cell renal cell carcinoma (ccRCC) significantly influenced survival rate of ccRCC patients. Here, we intended to investigate the impacts of circular RNA ITCH (circ-ITCH) on the metastasis of ccRCC. The expression of circ-ITCH in ccRCC tissues and cells was evaluated utilizing qRT-PCR. Transwell assay and wound healing were applied to investigate migration and invasion of ccRCC cells. Target gene prediction and screening and luciferase reporter gene assays were utilized to assess downstream target genes of circ-ITCH. Western blot was utilized to detect metastasis-related protein expression. A xenograft tumor model was established to evaluate the role of circ-ITCH in vivo. Results showed that circ-ITCH was low expressed in ccRCC tissues and cells. Downregulation circ-ITCH promoted cell migration, but overexpressing circ-ITCH inhibited cell migration and invasion in OSRC-2 and SW839 cells. Mechanism investigations claimed that circ-ITCH exerted its metastasis-inhibitory activity via sponging miR-106b-5p and regulating the expression of PDCD4. Conclusively, circ-ITCH suppresses ccRCC metastasis by enforcing PDCD4 expression through binding miR-106b-5p. circ-ITCH may function as a novel diagnostic target to suppress ccRCC metastasis.

## 1. Introduction

Clear cell renal cell carcinoma (ccRCC), the most common renal cell carcinoma (RCC) [1], characterizes with high metastasis [2, 3]. Therefore, the 5-year survival rate of RCC patients who are diagnosed at metastatic stage only have 12% [4]. At present, it lacks targeted and effective management marker for ccRCC metastasis in clinic. There is an urgent need to search for novel diagnosis target for ccRCC metastasis.

Circular RNAs (circRNAs) are particular type of noncoding RNA molecule that featured with closed circular structure and stability [5]. circRNAs are associated with proliferation, invasion, and metastasis in cancers including colorectal cancer, lung cancer, bladder cancer, pancreatic cancer, non-small-cell lung cancer, and endometrial carcinoma [6–9]. In RCC, previous studies showed that circ-AKT3 [10], cRAPGEF5 [11], circ_000926 [12], hsa-circ-0072309 [13], circ-EGLN3 [14], circ-ZNF652 [15], has_circ_0054537 [16], circTLK1 [17], circMTO1 [18], circ-0001368 [19], circPRRC2A [20], and has_circ_0035483 [21] were involved in cancer progression. However, there is no substantive progress in the RCC treatment yet. So it is necessary for RCC to find new circRNAs target. Among these circRNAs, circular RNA ITCH (circ-ITCH) acts as a tumor suppressor in the occurrence of several tumors, such as hepatocellular carcinoma [22], ovarian cancer [23], oral squamous cell carcinoma [24], cervical cancer [25], osteosarcoma [26], and prostate cancer [27]. But the role of circ-ITCH in ccRCC is not clarified now.

Programmed cell death 4 (PDCD4), a tumor suppressor, is firstly clarified as a gene that induces apoptosis in murine cell line [28]. It is frequently downregulated in cancers and inhibits tumor promotion, development, proliferation, invasion, and metastasis [29, 30]. Studies showed that PDCD4 was downregulated in RCC patients and strongly associated with tumor stage, tumor grade, tumor metastasis, and tumor-related death [31]. However, the corresponding mechanism in RCC is not understood clearly. Hao et al. [30] found that circular RNA ITCH could regulate PDCD4 to inhibit cell proliferation and promote cell apoptosis in oral squamous cell carcinoma. Whether circ-ITCH and PDCD4 control ccRCC development is not reported.

In this study, we examined the level of circ-ITCH in ccRCC tissues and cells and found that circ-ITCH was downexpressed in ccRCC. Further studies were conducted to explore the function of circ-ITCH in ccRCC by utilizing the ccRCC cell lines and xenograft model. Systematic studies revealed that circ-ITCH hindered ccRCC migration and invasion by sponging miR-106b-5p to regulate PDCD4. Consequently, circ-ITCH might become a novel target to inhibit ccRCC metastasis.

## 2. Materials and Methods

### 2.1. Tissue Samples

Fifty-four pairs of ccRCC tissues and paired adjacent normal kidney tissues were collected for research in this work; the characteristic information of samples were listed in supplementary file (Table S1). The tissue samples were quickly frozen in liquid nitrogen after surgery and stored until use. Histological and pathological diagnoses of the specimens were confirmed according to the 2016 World Health Organization Consensus Classification and Staging System of Renal Tumor and Fuhrman grade by two experienced pathologists. All specimens were obtained with informed consent of the patients and approved by the Ethics Committee of the Affiliated Huai'an Hospital of Xuzhou Medical University.

### 2.2. Cell Lines and Culture

Human normal kidney cell line HK-2 and ccRCC cell lines OSRC-2, A498, SW839, 786-O, Caki-1, and GRC-1 were purchased from the American Type Culture Collection (Shanghai, China). All cell lines were cultured in RPMI-1640 medium, which contained 10% fetal bovine serum (FBS) and 1% penicillin/streptomycin (Gibco, USA). Cells were cultured in CO_2_ incubator (37°C, 5% CO_2_).

### 2.3. Cell Transfection

Overexpression vector or downexpression vector of circ-ITCH was acquired by cloning the sequence of circ-ITCH into the pcDNA vector (Shanghai, China). miR-106b-5p mimic, miR-106b-5p inhibitor, si-PDCD4, OE-PDCD4, and their respective controls were purchased from RiboBio (Guangzhou, China). OSRC-2 and SW839 cells were transfected using lipofectamine 2000 (Invitrogen, USA).

### 2.4. Transwell Assay

Cell migration and invasion were performed by Transwell chambers (BD Biosciences, USA). After 48 h transfection of plasmid, OSRC-2 and SW839 cells were resuspended in 200 *μ*L serum-free RPMI-1640 medium with the density of 3 × 10^5^, then were placed in the upper chamber with 8 *μ*m pore filters (Millipore, Germany). And RPMI-1640 medium with 10% FBS was added to the lower chambers. After incubation, cells were washed three times with PBS and handled by utilizing the methanol to fix and crystal violet to stain (Gibco, USA). Finally, cell photos were graphed under the microscope (Canon, Japan) at ×200 magnification.

### 2.5. Wound Healing

Cell migration was also tested by wound healing assay. OSRC-2 and SW839 cells (3 × 10^5^ cells per well) separately were seeded into six-well plates. And lines were drawn on the back of the plate via marker pens. When cells covered the plate surface, the cells were treated overnight with 1% FBS. Then, 200 mL sterile pipette tips were used to draw a straight line on the cell surface. The fallen cells were washed with 2 mL of PBS for three times. After that, the cells were cultured with serum-free medium in CO_2_ incubator (37°C, 5% CO_2_). After 24 hours, the cells were photographed with the ImageJ software.

### 2.6. Luciferase Report Assay

The luciferase reporter vector pmirGLO was used to clone the full length of 3′-UTR of circ-ITCH and PDCD4. Then, the recombination plasmid (circ-ITCH-WT or circ-ITCH-MUT, PDCD4-WT or PDCD4-MUT) was transfected with miR-106b-5p mimic or miR-106b-5p NC into OSRC-2 and SW839 cells separately. And cells were incubated for 48 h. Then, the luciferase activities were analyzed via dual-luciferase reporter assay (Promega, USA).

### 2.7. Quantitative Real-Time PCR

Total RNA was isolated by using TRIzol reagent (Invitrogen, Canada). Reverse transcription was conducted by Transcriptor First Strand cDNA Synthesis Kit (Roche Diagnostics, Switzerland). Real-time PCR was operated using SYBR ® Green PCR Kit (Qiagen). The expression levels of the fold changes of the genes were calculated by 2^−*ΔΔ*Ct^ method and normalized with GAPDH (5′GGAGCGAGATCCCTCCAAAAT3′, 5′GGCTGTTGTCATACTTCTCATGG3′).

### 2.8. Western Blot

RIPA Lysis Buffer (HY-K1001, MedChem Express) was used to separate total protein in cells. The protein was transferred onto PVDF membranes (Millipore, MA), which were then blocked with skim milk for 1 h at room temperature. Else, the primary antibodies (anti-GAPDH, anti-E-cadherin, anti-N-cadherin, anti-PDCD4) were added for overnight incubation, followed by HRP-conjugated secondary antibody. Protein bands were analyzed by the ImageJ software.

### 2.9. In Vivo Studies

12 eight-week old male BALB/c mice were used to establish ccRCC-bearing xenograft models and were randomly divided into the NC group and OE-circ-ITCH group. Almost 3 × 10^5^ OE-NC or OE-circ-ITCH RCC cells were injected into the armpits of nude mice. Mice were sacrificed after 8 weeks, and tumor width and length were measured by digital calipers. The tumor volumes were calculated using the formula: volume = (length × width^2^)/2. Tumor weights were collected after mice were sacrificed.

### 2.10. Statistical Analysis

Data analysis was carried out by the OriginPro 9 and the GraphPrism software. The data is presented as the mean ± SD. Group differences were tested for statistical significance using Student's *t*-test. *p* < 0.05 represents a significant difference. For each group, there were at least three replicates.

## 3. Results

### 3.1. circ-ITCH Expression Is Downregulated in ccRCC

**Figure 1:**
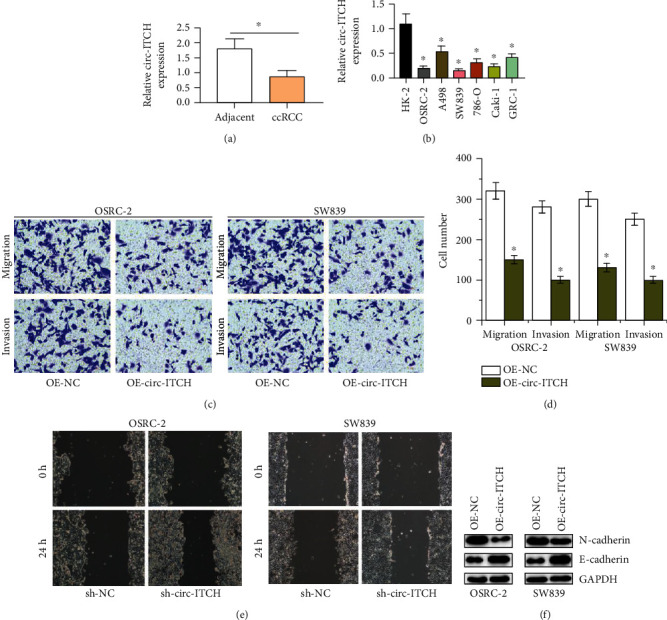
circ-ITCH inhibits ccRCC cell migration and invasion. (a, b) The expression of circ-ITCH in ccRCC tissues and cells by qT-PCR. (c, d) Transwell assay determines the role of circ-ITCH in migration and invasion of OSRC-2 and SW839 cells. (e) Wound healing detects the effect of circ-ITCH downregulation on the migration abilities of OSRC-2 and SW839 cells. (f) The expressions of E-cadherin and N-cadherin in OSRC-2 and SW839 cells transfected with OE-circ-ITCH.

We firstly detected the circ-ITCH expression in ccRCC tissues and cells by qRT-PCR. The results are shown in Figures 1(a) and 1(b); circ-ITCH levels were significantly reduced in ccRCC tissues than those in nearby normal tissues. And circ-ITCH levels in ccRCC cell lines were significantly downregulated comparing with normal kidney cell line. Above results show that circ-ITCH is downregulated in ccRCC.

### 3.2. circ-ITCH Inhibits Migration and Invasion of ccRCC Cells In Vitro

Then, to explore the effects of the circ-ITCH dysregulation on the ccRCC metastasis, we constructed circ-ITCH-overexpressing OSRC-2 and SW839 cell lines. Transwell assay showed that the overexpression of circ-ITCH inhibited the abilities of migration and invasion in OSRC-2 and SW839 cells, as shown in Figures 1(c) and 1(d). Wound healing assay showed that circ-ITCH knockdown promoted cell migration in RCC (Figure 1(e)). And the metastasis associated protein E-cadherin expressions were upregulated, and the N-cadherin expressions were downregulated in circ-ITCH-overexpressing ccRCC cells in Figure 1(f). These data suggest that circ-ITCH could inhibit migration and invasion of ccRCC cells.

### 3.3. circ-ITCH Acts as a Sponge for miR-106b-5p in ccRCC Cells

**Figure 2:**
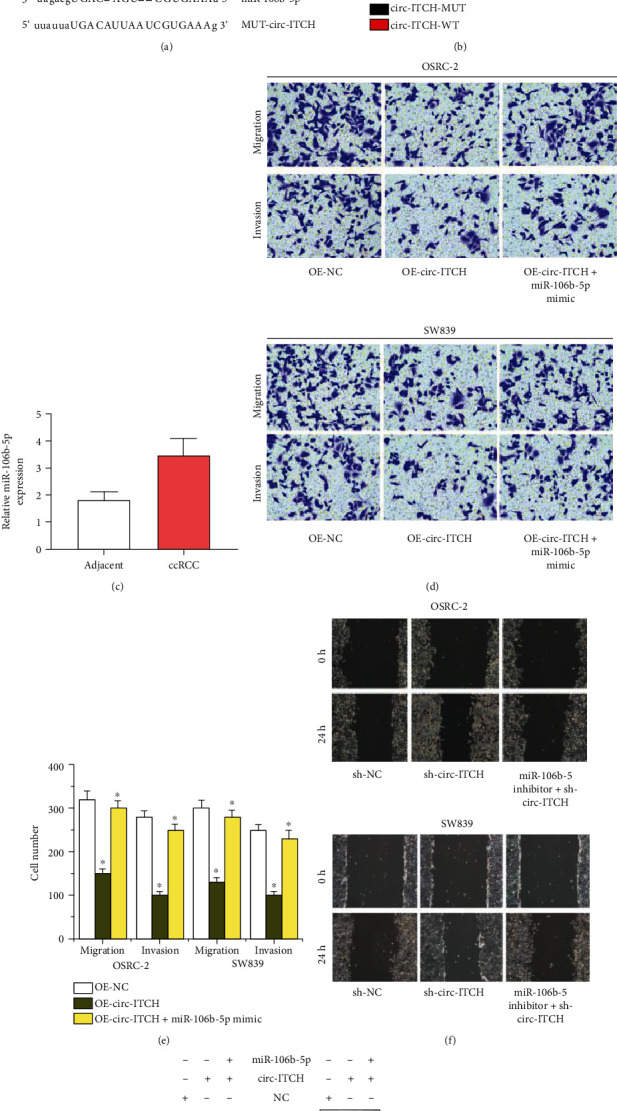
circ-ITCH inhibits ccRCC cell metastasis via sponging miR-106b-5p. (a) The predicted binding sites between circ-ITCH and miR-106b-5p. (b) The dual luciferase reporter assay detects the luciferase activity of circ-ITCH-WT/circ-ITCH-MUT in OSRC-2 and SW839 cells cotransfected with miR-106b-5p mimics. (c) The expression of miR-106b-5p in ccRCC tissues. (d, e) Transwell assay detects migration and invasion abilities of OSRC-2 and SW839 cells transfected with OE-NC, OE-circ-ITCH, OE-circ-ITCH, and miR-106b-5p mimic. (f) Wound healing detects migration abilities of OSRC-2 and SW839 cells transfected with sh-NC, sh-circ-ITCH, and sh-circ-ITCH + miR-106b-5p inhibitor. (g) Western blot analyzes the expressions of E-cadherin, N-cadherin in OSRC-2, SW839 cells transfected with NC, OE-circ-ITCH, OE-circ-ITCH, and miR-106b-5p mimic.

To study the mechanism of circ-ITCH mediated the migration and invasion of ccRCC cells, we applied the miRNA target prediction tool (StarBase Database, http://starbase.sysu.edu.cn/). Firstly, we identified the potential binding sites for circ-ITCH and miR-106b-5p in Figure 2(a). Then, we found that luciferase activity was significantly reduced in ccRCC cells stimulated with circ-ITCH-WT and miR-106b-5p, but this effect did not exist in ccRCC cells stimulated with circ-ITCH-MUT and miR-106b-5p, as shown in Figure 2(b). Furthermore, we measured the expression of miR-106b-5p in ccRCC tissues and noticed that miR-106b-5p was significantly upregulated in ccRCC tissues in Figure 2(c). Subsequently, functional studied showed that inducing the expression of miR-106b-5p would reverse the inhibitory effect of circ-ITCH overexpression on the migration and invasion abilities of ccRCC cells in Figures 2(d) and 2(e). Wound healing in Figure 2(f) showed that circ-ITCH knockdown promoted RCC cells migration, but extra miR-106b-5p inhibitor supplement in RCC cells transfected with sh-circ-ITCH would inhibited cell migration compared with RCC cells transfected with sh-circ-ITCH. The expressions of E-cadherin and N-cadherin were reversely regulated by promoting the miR-106b-5p expression in circ-ITCH overexpressing OSRC-2 and SW839 cells than that of circ-ITCH overexpressing OSRC-2 and SW839 cells, as shown in Figure 2(g). These findings indicate that circ-ITCH sponges miR-106b-5p in ccRCC cells to regulate cell metastasis.

### 3.4. miR-106b-5p Directly Targets PDCD4 in ccRCC Cells

**Figure 3:**
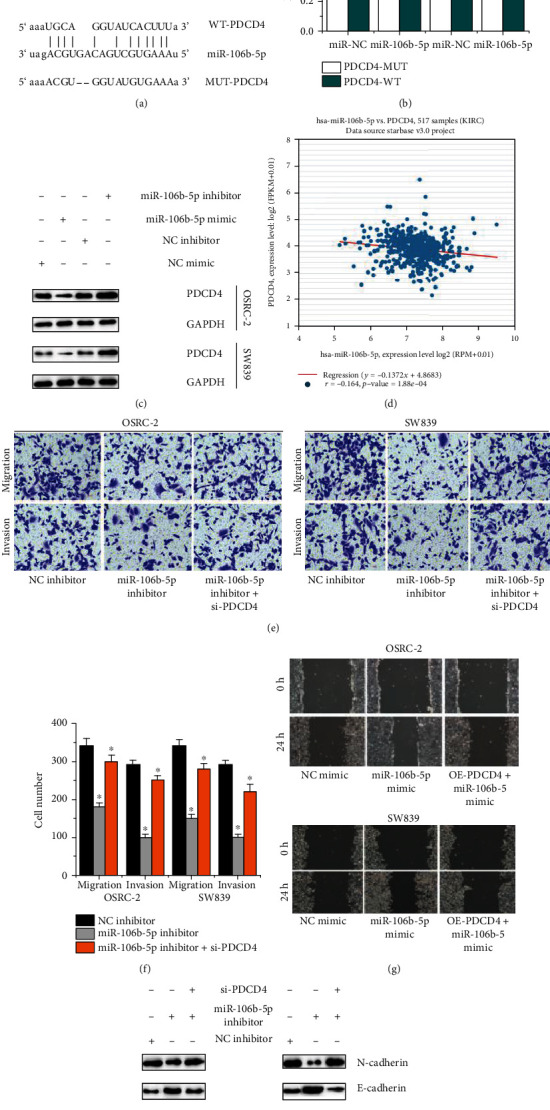
PDCD4 mediates miR-106b-5p-dependent ccRCC cell migration and invasion. (a) The predicted binding sites between miR-106b-5p and PDCD4. (b) The dual luciferase reporter assay detects the luciferase activity of PDCD4-WT/PDCD4-MUT in OSRC-2 and SW839 cells cotransfected with miR-106b-5p mimics. (c) The relation of the miR-106b-5p expressions and PDCD4 expressions in renal cancer. (d) Western blot determines the PDCD4 expressions in OSRC-2 and SW839 cells transfected with miR-106b-5p mimics and miR-106b-5p inhibitor. (e, f) Transwell assay detects migration and invasion abilities OSRC-2 and SW839 cells transfected with NC inhibitor, miR-106b-5p inhibitor, si-PDCD4, and miR-106b-5p inhibitor. (g) Wound healing detects migration abilities of OSRC-2 and SW839 cells transfected with NC mimic, miR-106b-5p mimic, OE-PDCD4, and miR-106b-5p mimic. (h) Western blot analyzes the expressions of E-cadherin, N-cadherin in OSRC-2, SW839 cells transfected with NC inhibitor, miR-106b-5p inhibitor, si-PDCD4, and miR-106b-5p inhibitor.

According to the StarBase database, PDCD4 was predicted as a target for miR-106b-5p in Figure 3(a). By dual luciferase reporting test, we found that luciferase activity was significantly reduced in ccRCC cells stimulated with PDCD4-WT and miR-106b-5p, but there were no evident differences in ccRCC cells transfected PDCD4-MUT and miR-106b-5p, as shown in Figure 3(b). In renal cancer patients, it was negatively related between the PDCD4 expressions and the miR-106b-5p expressions (Figure 3(c)). Western blot also showed that ccRCC cells transfected with miR-106b-5p mimic inhibited the PDCD4 expression, but ccRCC cells transfected with miR-106b-5p inhibitor induced the PDCD4 expression, as shown in Figure 3(d). Furthermore, Transwell assay suggests that the inhibition of miR-106b-5p would inhibit the migration and invasion of ccRCC cells. However, PDCD4 knockout in ccRCC cells transfected with miR-106b-5p inhibitor could induce the migration and invasion of ccRCC cells compared with ccRCC cells transfected with miR-106b-5p inhibitor, as shown in Figures 3(e) and 3(f). Wound healing showed that miR-106b-5p overexpression promoted RCC cells migration, but extra PDCD4 overexpression supplement in RCC cells transfected with miR-106b-5p mimic would inhibited cell migration compared with RCC cells transfected with miR-106b-5p mimic, as shown in Figure 3(g). Else, the miR-106b-5p inhibition induced the E-cadherin expression and inhibited the N-cadherin expression in ccRCC cells. However, PDCD4 knockout in ccRCC cells transfected with miR-106b-5p inhibitor could exerted an opposite effect on the E-cadherin and N-cadherin expressions compared with ccRCC cells transfected with miR-106b-5p inhibitor in Figure 3(h). Above results show that miR-106b-5p targets PDCD4 to mediate ccRCC cell migration and invasion.

### 3.5. circ-ITCH Overexpression Restrained the Growth of ccRCC Cells In Vivo

**Figure 4:**
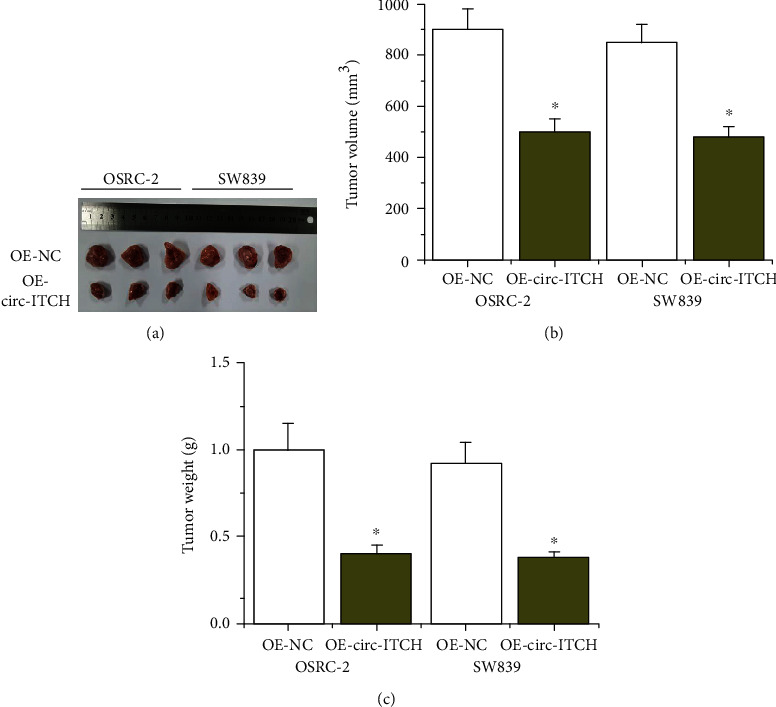
circ-ITCH exerts oncogenic effects of ccRCC cells in vivo. (a–c) Tumor specimens, volume, and weight in nude mice that injected with OE-circ-ITCH cells.

To verify the role of circ-ITCH in vivo, we established a xenograft tumor mouse model of ccRCC. As shown in Figures 4(a)–4(c), the overexpression of circ-ITCH would significantly decrease the tumor volume and tumor weight. The data provide that circ-ITCH overexpression could inhibit the development of ccRCC.

## 4. Discussion

circRNAs are reported to play important roles in taking part in tumorigenesis. As a common tumor suppressor in multiple cancers [22–27], the function of circ-ITCH in ccRCC is not clear. In this research, we firstly found that circ-ITCH levels were reduced in ccRCC tissues and ccRCC cell lines, which was similar with the fact that it existed the abnormal expression of circ-ITCH in other cancers [32]. And we observed that the downregulation of circ-ITCH promoted RCC cell migration. However, the upregulation of circ-ITCH suppressed the migration and invasion of ccRCC cell lines, decreased the expression of N-cadherin, and boosted the expression of E-cadherin.

To explore how circ-ITCH functioned in ccRCC, the deep investigations and research were operated. circRNAs can sponge miRNAs to regulate the mRNA expression in cancers, which is involved in the occurrence of cancers [33, 34]. We achieved the predicting binding sites between circ-ITCH and miR-106b-5p. By dual luciferase report assay, it is verified that miR-106b-5p coculture with ccRCC cells decreased the luciferase activity of circ-ITCH-WT not circ-ITCH-MUT. Different with the findings that circ-ITCH was downregulated in ccRCC tissues, miR-106b-5p was upregulated in ccRCC tissues. And circ-ITCH could mediate ccRCC cell migration and invasion via regulating miR-106b-5p. In addition, we found that miR-106b-5p negatively regulated PDCD4 in ccRCC cells and renal cancer patients. miR-106b-5p regulated ccRCC cell migration and invasion by targeting PDCD4. In previous works, PDCD4 was reported to be reduced in human renal cell carcinoma patients [27]. miR-21 downregulated PDCD4 to contribute to the RCC progression [35–37], which was similar with our findings that PDCD4 targeted miR-106b-5p to promote the ccRCC development. In vivo, we overexpressed circ-ITCH in BALB/c mice stimulated with ccRCC cells and noticed that the growth of tumors was then inhibited.

In this work, it reports the role of circ-ITCH in ccRCC migration and invasion. However, it still exists some limitations, such as small sample size, the unclear clinical diagnose effect of circ-ITCH in ccRCC metastasis. Subsequently, the samples of ccRCC patients will be increased to study the expression of circ-ITCH. It is worth exploring the relationship between circ-ITCH expression and tumor metastasis levels in ccRCC patients.

In conclusion, we demonstrated that circ-ITCH was a tumor suppressor in ccRCC via miR-106b-5p/PDCD4 pathway. It announced the novel molecular basis of circRNAs in the research of ccRCC metastasis.

## Data Availability

I confirm that I have included a citation for available data in my references section.

## Supplementary Materials

Supplementary Materials Clinical characteristics of 44 ccRCC patients in this work was listed in Table S1 in uploaded supplementary file.
