# Supplementary material for: Circular RNA ITCH Is a Tumor Suppressor in Clear Cell Renal Cell Carcinoma Metastasis through miR-106b-5p/PDCD4 Axis
Source: J Immunol Res. 2021 Apr 20;2021:5524344. doi: 10.1155/2021/5524344 (PMC8081612; doi:10.1155/2021/5524344)
Supplement: Supplementary Materials — Clinical characteristics of 44 ccRCC patients in this work was listed in Table S1 in uploaded supplementary file. [file 5524344.f1.pdf]

Table S1. Clinical characteristics of 44 ccRCC patients

**Variables**	
**Gender**	
Male	n=28
Female	n=16
**Age (years)**	
<60	n=30
≥60	n=14
**Tumor size (cm)**	
<4	n=17
≥4	n=27
**Grade**	
Well differentiated (grade 1 and 2)	n=18
Moderately differentiated (grade 3)	n=18
Poorly differentiated (grade 4)	n=8
**Metastasis**	
Yes	n=33
No	n=11
